# Delineating Oxidation Aspects of an Additively Manufactured Nanoprecipitation-Strengthened Al_0.2_Co_1.5_CrFeNi_1.5_Ti_0.3_ High-Entropy Alloy

**DOI:** 10.1021/acsanm.6c00394

**Published:** 2026-06-02

**Authors:** Poresh Kumar, Tu-Ngoc Lam, Po-Heng Chou, An-Chou Yeh, Peter K. Liaw, E-Wen Huang, Sudhanshu Shekhar Singh

**Affiliations:** † International College of Semiconductor Technology, 34914 National Yang Ming Chiao Tung University, Hsinchu 300093, Taiwan; ‡ Department of Materials Science and Engineering, National Yang Ming Chiao Tung University, Hsinchu 300093, Taiwan; § Department of Materials Science and Engineering, Indian Institute of Technology, Kanpur 208016, India; ∥ High Entropy Materials Center, 34881 National Tsing Hua University, Hsinchu 300044, Taiwan; ⊥ Cuu long University, Vinh Long Province, 890000, Vietnam; # Department of Materials Science and Engineering, National Tsing Hua University, Hsinchu 300044, Taiwan; ∇ Department of Materials Science & Engineering, The University of Tennessee, Knoxville 37996-2100, United States

**Keywords:** high-entropy alloys, additive manufacturing, oxidation, oxide-film growth kinetics, precipitation strengthening

## Abstract

The recent perspective of multiprincipal element alloys (MPEAs), also known as high-entropy alloys (HEAs), has emerged as a very promising area for material design. Additive manufacturing (AM) strategies have also been noted to provide additional strength to HEA systems. In the selected dual-nanoprecipitation Al_0.2_Co_1.5_CrFeNi_1.5_Ti_0.3_ HEA system, an additional strength of approximately 300–400 MPa was achieved by adopting an additive manufacturing route as compared to its cast and wrought counterparts. However, the challenge of oxidation degradation always imposes a severe limitation for high-temperature applications in gas turbines, power plants, and aerospace components. Hence, ensuring material sustainability, longevity, and integrity for high-temperature applications inevitably requires the exploration of the oxidation behavior of alloys. In the current study, the oxidation performance of Al_0.2_Co_1.5_CrFeNi_1.5_Ti_0.3_ HEA, in as-printed as well as nanoprecipitation-strengthened aged states, was evaluated from 600°C to 1200°C. A comparative framework elucidating the mechanistic aspects and elemental redistribution of nanoprecipitates on oxidation behavior has been highlighted. In both the as-printed and aged states, the alloys followed subparabolic oxidation weight gain kinetics below 900°C. However, the thickness growth kinetics exhibited parabolic behavior above 900°C. The oxide layer exploration manifested the formation of a homogeneous Cr-oxide layer, which acts as a protective barrier against oxidation activity. The impact of atomic size on mobility also played a significant role in suppressing the formation of outer Al and Ti oxide layers, instead of having a lower reduction potential compared to Cr.

## Graphical abstract

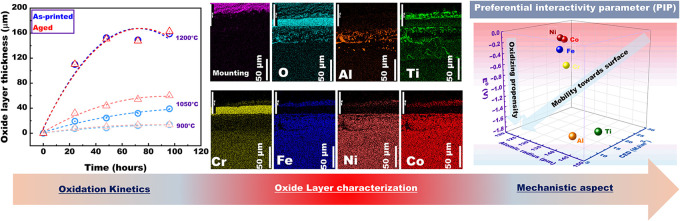

## 1 Introduction

The design and development of high-performance alloys with sustainability at elevated temperatures and in harsh environments have always been of keen interest to the scientific community. Thermodynamically, the high-temperature sustainability of materials requires energy efficiency and economic significance. Over time, a new alloy design scheme was introduced, called multiprincipal element alloys (MPEAs). The concept of MPEAs was first introduced by Yeh et al.^1^ and Cantor et al.^2^ upon the observation of simple solid solution phases in multimetallic mixtures. The evolution and stability of simple solid solution phases were hypothesized based on the high configurational entropy effect, and hence, the term high-entropy alloy (HEA) was coined for this class of alloy systems. Concurrently, other core effects, such as severe lattice distortion, sluggish diffusion, and cocktail synergies due to compositional complexities, have also been proposed. Since these systems consist of multiple principal elements, they facilitate huge opportunities for alloy design through elemental as well as compositional variations. Owing to their immense design possibilities, HEAs have been extensively explored for their mechanical and functional properties. The research findings reported in the literature demonstrate impressive properties, such as superior mechanical attributes,^3–7^ wear and corrosion resistance,^8–10^ and excellent thermal stability.^11^ To achieve significant advancements in mechanical properties, multiphase and nanoprecipitation strengthening strategies have also been employed.^12–14^ Tailoring phase evolution and acquiring precipitation by the partial addition of elements is the latest research trend in the HEA field.^15,16^ Several types of precipitates have been observed in HEAs.^17^ However, L1_2_-type nanoprecipitates, which are model precipitates in superalloys, have been the major focus in HEAs.^18^ The effects of Al and Ti additions on the characteristics of L1_2_ types of precipitates in CoCrFeNi-based transition-metal-based HEA systems have been explored.^9,19,20^ An Al- and Ti-optimized Al_0.2_Co_1.5_CrFeNi_1.5_Ti_0.3_ HEA system was proposed, with homogeneously distributed L1_2_ nanoprecipitates with considerable mechanical attributes.^15,19^ The Co and Ni contents shifted toward the higher side to acquire the matrix as fcc, which renders formability.^21,22^ Moreover, emerging additive manufacturing (AM), also known as 3D printing, facilitates microstructural refinement and property enhancement, along with geometrical design flexibility.^23–27^ Upon applying the selective laser melting (SLM)-based AM technique on the Al_0.2_Co_1.5_CrFeNi_1.5_Ti_0.3_ HEA system, a significant improvement in strength was observed.^28^ Consistent with this, our recent investigation on the high-cycle and low-cycle fatigue behaviors of the same alloy also demonstrates notable endurance strength and fatigue life, respectively, as compared with conventional alloys.^29,30^ The observed improvement was attributed to the AM-induced microfeatures, such as higher dislocation density, cell structures, as well as additional L2_1_-type nanoprecipitates under aging conditions.^28^ However, industrial environments and high-temperature exposure to oxidizing/reducing gases cause material degradation, leading to catastrophic failures. Hence, the practical feasibility, longevity, and sustainability of materials for high-temperature applications merit the exploration of oxidation aspects at elevated temperatures. In recent times, understanding the oxidation behavior of HEAs has emerged as a trending research area.

Since the introduction of the HEA concept, aiming for high-temperature compatibility, several oxidation studies at elevated temperatures have been conducted on HEA systems.^31–35^ Following the traditional strategy of achieving a dense oxide layer on the surface to suppress oxygen and metal cation diffusion,^36,37^ oxidation studies were conducted on some selected HEAs consisting of sufficient amounts of Al, Cr, and Si.^38–40^ These elements form dense protective oxide layers and effectively suppress the oxidation of the base alloy. The partial addition of these elements has mostly been incorporated in refractory HEAs (RHEAs) because they are bcc stabilizers. Liu et al.^41^ investigated the elemental as well as combined effect of Ti, V, and Si addition on the RHEA system and demonstrated the beneficial impact of Ti–Si addition against V. Similarly, the impact of partial Al and Si addition on transition-metal-based HEAs was also investigated, highlighting the beneficial impact of Si.^42^ However, Si content up to a certain limit has a beneficial impact on oxidation resistance due to phase readjustment.^40^ The impact of Al addition on oxidation resistance was also highlighted from the perspective of phase readjustment due to the Al content and processing.^43,44^ To date, most of the oxidation investigations on HEAs have focused on the elemental impact, and the entire investigation has concentrated on the formation of protective oxide scales, which suppress the diffusion of oxygen and metal cations.^31,43,45,46^ Unfortunately, due to the synergistic effect of the elements and the inherent sluggish diffusion characteristics, the oxidation process is very complex in HEAs. The oxide products consist of various complex oxides as well as spinel oxides.^31,32,34,35^ Recently, Lee et al.^35^ investigated the oxidation behavior of 3D-printed L1_2_ nanoprecipitation-strengthened HEAs and highlighted the formation of complex oxides. Hence, understanding the oxidation process, elucidating the layered growth of the oxide scale, and determining the role of nanoscale interfaces are critical for ensuring material integrity at elevated temperatures.

Motivated by the outstanding mechanical attributes such as the tensile and fatigue properties of the 3D-printed nanoprecipitation-strengthened Al_0.2_Co_1.5_CrFeNi_1.5_Ti_0.3_ HEA,^29,30^ in the current study, we have explored the oxidation behavior emphasizing high-temperature application compatibility and long-term service performance in gas turbines, power plants, and aerospace sectors. A detailed scheme of the study is presented in Figure 1. Although previous work by Yang et al.^46^ on the same alloy system processed via conventional casting reported the formation of a protective Cr_2_O_3_ layer, their study was limited to a single temperature and focused on the comparative effect of Nb addition. Furthermore, the differences in the microstructural and mechanical characteristics between the conventionally processed (with only a single L1_2_ nanoprecipitate) and additively manufactured states (with both L1_2_ + L2_1_ nanoprecipitates) necessitate a more comprehensive evaluation. Accordingly, isothermal oxidation experiments were conducted over a wide temperature range from 600°C to 1200°C, and both weight gain and oxide growth kinetics were investigated. The oxide scale characterization and the mechanistic aspects of oxide scale formation are highlighted. The observed results indicate a shift from subparabolic to parabolic oxidation kinetics with increasing temperature. The formation of a homogeneous Cr-oxide layer hinders oxygen and element diffusion and hence provides oxidation resistance. Thermodynamics and diffusion kinetics control the formation of inhomogeneous Al and Ti oxide layers beneath the Cr-oxide layer. The larger atomic size, as well as relatively lower concentrations of Al and Ti, restricted their mobility. In addition, diffusion-related factors such as the activation energy and ionic valence further limit their mobility, thereby preventing the formation of a continuous outer Al and Ti oxide layer, despite their comparatively lower standard reduction potentials (higher oxidizing tendencies) than Cr.

**Figure 1:**
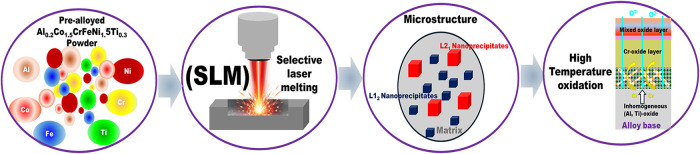
Schematic illustration of the alloy composition, additive manufacturing process, nanoscale microstructure evolution, and oxidation scale formation.

## 2 Experimental Details

### 2.1 Alloy Preparation and Initial Characterization

Al_0.2_Co_1.5_CrFeNi_1.5_Ti_0.3_ alloys were prepared from a prealloyed powder using an SLM-based AM technique. A zigzag scanning strategy with a 67° rotation in successive layers was employed to build the sample, as described in Figure 2. The alloy was printed on a heated plate using a laser power of 260 W, 58 μm spot size, 70 μm hatch spacing, and 1000 mm/s scanning speed on 50 μm powder layers under an argon atmosphere. These parameters were chosen based on prior studies, indicating optimization to obtain the highest density (higher than 97%) and limited porosity.^28^ In line with our aim of study, the printed samples were removed from the plate, and some samples were directly aged at 750°C for 50 h to achieve optimum precipitation, which results in precipitation strengthening.^28,29^ Further, the initial microstructure was investigated using scanning electron microscope (SEM) and neutron diffraction methods. To investigate the initial microstructure using SEM, samples were ground, polished, and then electrochemically etched in 20 vol % phosphoric acid at 5 V for 15 s with a copper electrode.

**Figure 2:**
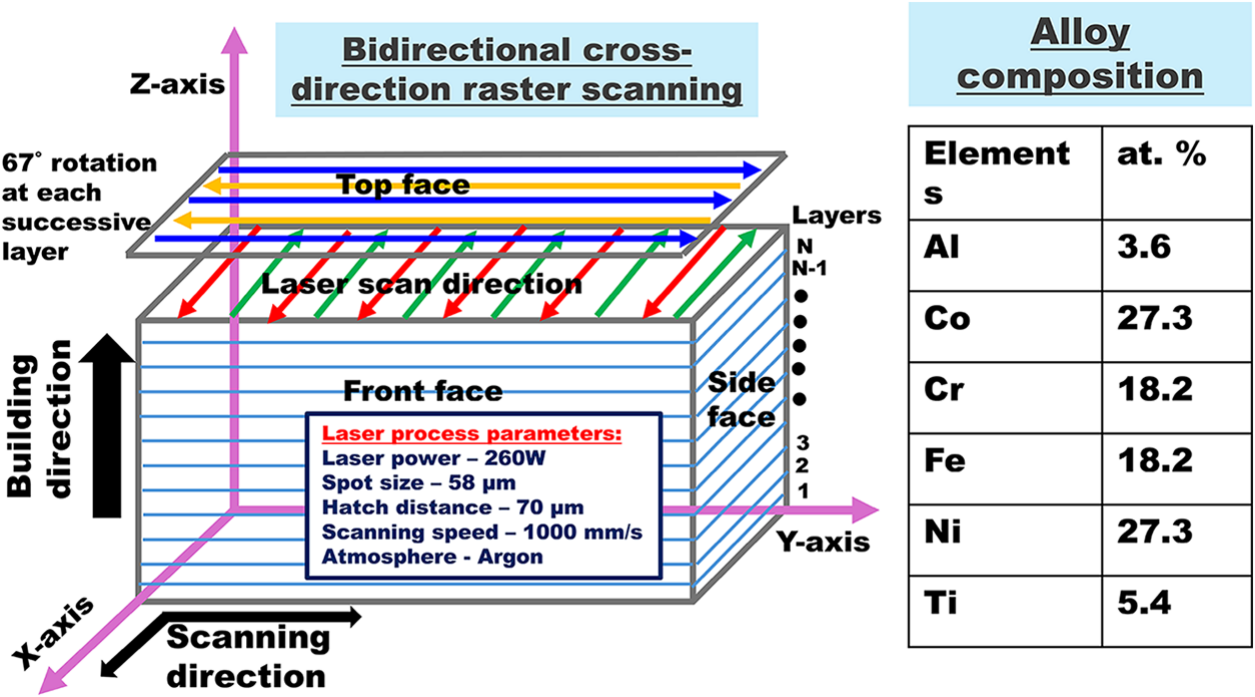
Schematic of laser printing strategies, laser printing parameters, and alloy composition (inset table).

### 2.2 Oxidation Tests

To investigate the oxidation behavior of the samples, the as-printed and aged samples were sectioned from the blocks. Samples with dimensions of 7.5 × 7.5 × 3mm^3^ were cut, and each face was polished up to 4000 grit paper. The polished samples were cleaned with alcohol and dried before the oxidation test. To investigate the oxidation weight gain and oxide growth kinetics, isothermal oxidation tests of the selected alloy in both the as-printed and aged conditions were conducted at 600°C, 750°C, 900°C, 1050°C, and 1200°C for 96 h. Oxidation tests at 500°C, 750°C, and 900°C were specifically conducted in a tubular furnace equipped with an integrated weighing machine, as shown schematically in Figure S1. Weight gain over a period of 96 h was recorded at intermittent time intervals using OBS Studio software to study the oxidation weight gain kinetics. To study the oxide growth kinetics, tests at higher temperatures, such as 900°C, 1050°C, and 1200°C, were conducted in a muffle furnace, without a weighing machine, for 24, 48, 72, and 96 h at each temperature. The growth of the oxide layer thickness with time and temperature, as well as the nature of the formed oxide layers, was explored, and the growth kinetics were evaluated.

### 2.3 Post-Oxidation Characterizations

The oxide layers of the oxidation-tested samples were investigated using scanning electron microscopy (SEM) and energy-dispersive spectroscopy (EDS). The types of oxides formed at the surface during the oxidation tests were identified by X-ray diffraction (XRD) and Raman spectroscopy.

## 3 Results and Discussion

### 3.1 Alloy Microstructure

The initial microstructure of both the as-printed and aged alloys was investigated using SEM and neutron diffraction. The microstructures in both the SLM as-printed condition and after direct aging treatment, characterized through SEM, are depicted in Figure 3 (a,b), respectively. The as-printed alloy exhibits a characteristic cellular structure inherent to additively manufactured materials, comprising densely populated dislocations at the cell walls and relatively less populated cell interiors. The corresponding neutron diffraction pattern presented in Figure 3 (c) (blue) consists of only the fcc phase. After aging treatment at 750°C for 50 h, it develops homogeneously distributed L1_2_-type smaller precipitates and L2_1_-type blocky precipitates at the cell wall regions, as reported previously.^28–30^ The corresponding phases are also detected in the neutron diffraction pattern, as shown in Figure 3 (c) (red). The volume fraction of nanoscale L1_2_ and L2_1_ precipitations in the aged alloy is approximately 25–30%.^28–30^ The L1_2_ nanoprecipitates are enriched in Ni and moderately enriched in Ti relative to the nominal composition listed in the inset table of Figure 2. In contrast, the larger L2_1_ nanoprecipitates are enriched in Al and Ti while being depleted in Cr and Fe.^29^

**Figure 3:**
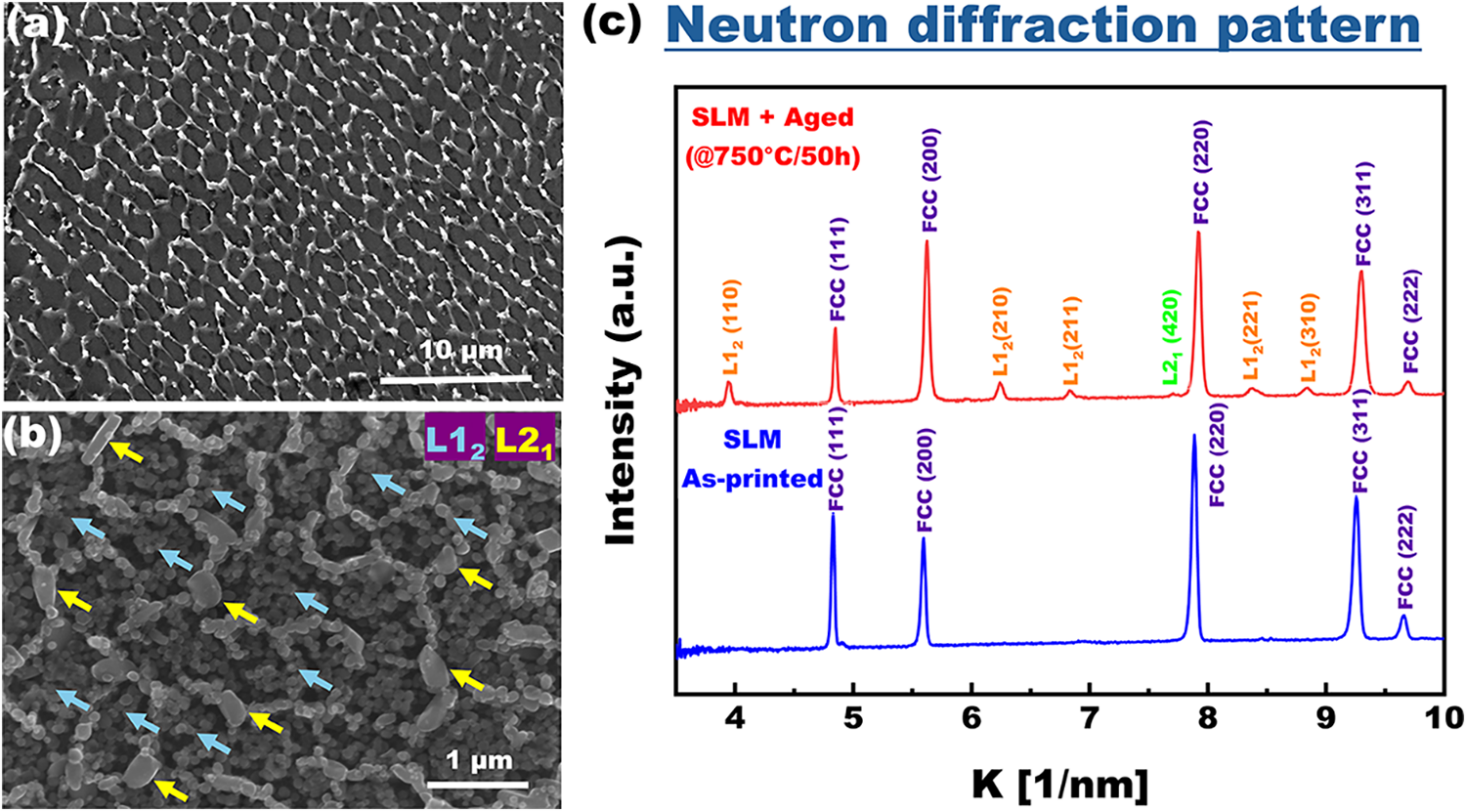
SEM micrographs of (a) SLM as-printed alloy; (b) SLM + aged alloy; (c) diffraction pattern of as-printed (blue) and aged alloys (red).

### 3.2 Oxidation Kinetics

The isothermal weight gain plots, demonstrating the oxidation kinetics with varying times at 600°C, 750°C, and 900°C, are presented in Figure 4 (a). The corresponding kinetic power law fittings using eq 1 ^47^ for both as-printed and aged alloys are shown in Figure 4 (b).

1 $$\left(\Delta w/A\right)=k_{p}t^{n}$$

where Δ*w* is the weight gain, *A* is the surface area of the sample, *k*_*p*_ and *t* denote the rate constant and time, respectively, and *n* is the time exponent, which determines the nature of the weight gain kinetics. The oxidation mass gain of the as-built alloy is minimal at 600°C and increases progressively with increasing temperature, indicating a temperature dependence of the oxidation kinetics. Similarly, a weight gain trend with temperature is also observed in the aged alloys. However, the weight gain is consistently higher than that of the as-printed alloy. Moreover, the increase in weight gain with temperature is more pronounced in the aged alloy. The weight gain plots for both alloys at the investigated temperatures exhibit a gradually decreasing rate of weight gain with exposure time. The corresponding time exponent (*n*) and rate constant (*k*_*p*_) obtained by fitting the data using eq 1 are tabulated in Table 1. The time exponent (*n*) for the as-printed alloy shows a decreasing trend with increasing temperature, whereas the aged alloy exhibits an initial decrease followed by an increase as the temperature increases. A time exponent value of 0.5 (*n* = 0.5) corresponds to parabolic kinetics, while <0.5 (*n* < 0.5) indicates subparabolic kinetics behavior.^48–50^ The variation in the time exponent with temperature reflects changes in the dominant oxidation mechanism and formed oxide scale characteristics. For both alloys at 600°C, the nearly parabolic behavior (*n* ∼ 0.5) suggests mixed control by the surface reaction and diffusion through a relatively unstable nonprotective oxide layer, as shown in Figure S2 (Visual camera image). At 750°C and 900°C, *n* < 0.5 indicates subparabolic kinetics, suggesting the formation of a dense and highly protective oxide scale that significantly restricts diffusion in the as-printed alloy. Similarly, in the aged alloy at 750°C, *n* < 0.5 indicates subparabolic kinetics, suggesting the formation of a dense and highly protective oxide scale. However, the kinetics transitioned to near-parabolic behavior (*n* ∼ 0.5), consistent with diffusion-controlled growth through a stable oxide scale, where enhanced diffusion at elevated temperatures governs oxidation. Over time, the oxidation behavior of traditional alloys, such as Ni- and Co-based superalloys, and HEAs at elevated temperatures (usually >500°C), has been extensively explored for high-temperature applications. These studies highlighted that the formation of specific elemental and complex oxides, along with their protective and adherence properties, governs oxidation resistance. These aspects are critical for alloy design from both thermodynamic and kinetic perspectives. In both the as-printed and aged conditions, the weight gain plot during the initial few hours shows a higher oxidation rate. Hypothetically, this suggests that all constituting elements have an equal probability of being oxidized in the initial period without any strong diffusion barrier due to the formation of loose and discontinuous oxides, such as NiO, CoO, Fe_2_O_3_, and TiO_2_.^51,52^ However, with time, the redox-driven elemental gradation dominates, and a specific protective oxide layer forms, suppressing the oxidation rate significantly. Such behavior, characterized by an initial stage of oxidation followed by preferential diffusion-controlled growth, has been reported in various HEAs.^46,53,54^ The observed higher weight gain kinetics variation with temperature in the aged alloy compared to the as-printed alloy is due to the inhomogeneous Cr distribution within the matrix and nanoprecipitate phases in the aged alloy.^28^ The nanoprecipitate phases in the aged alloy have a comparatively lower Cr concentration than the matrix, leading to the formation of a less protective Cr-oxide layer at the precipitates during exposure.^55,56^

**Figure 4:**
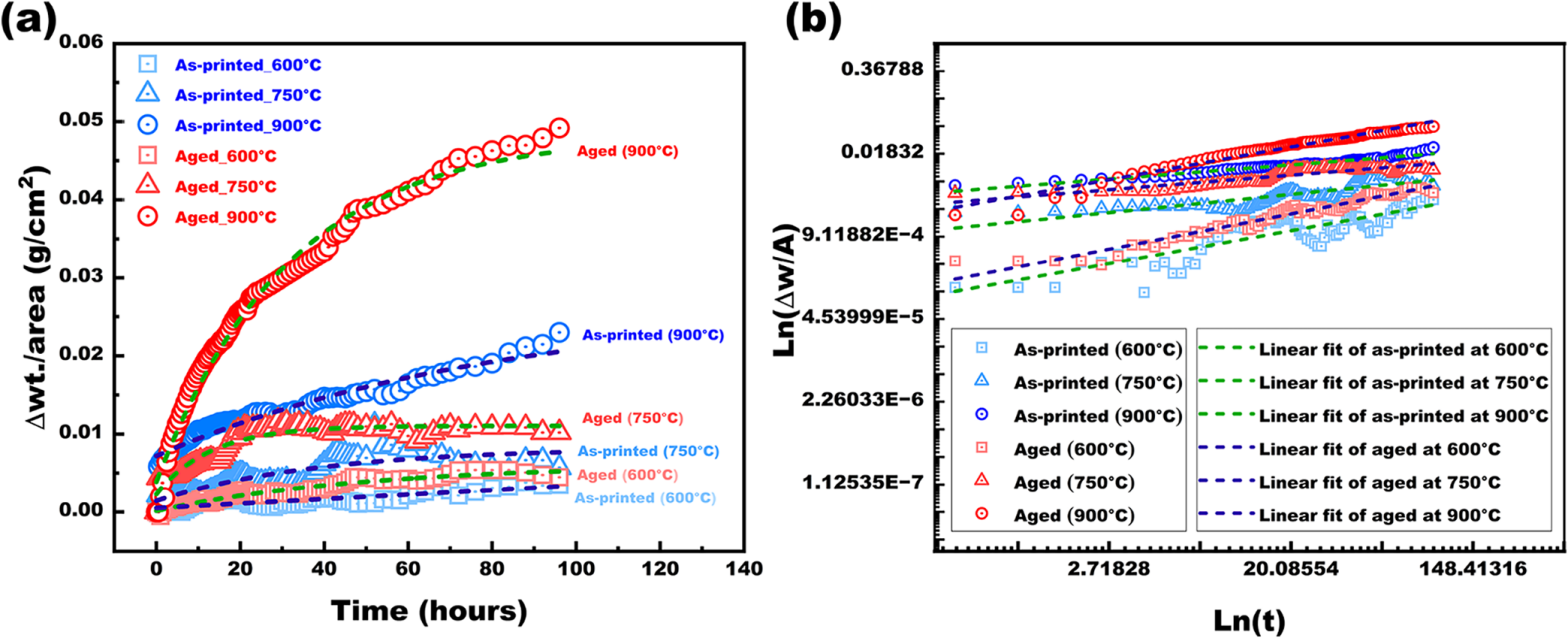
(a) Isothermal weight gain plot for the as-printed alloy (blue) and the aged alloy (red); (b) kinetic power law fitting {linear regression of eq 1} at different temperatures for the as-printed and aged alloys.

**Table 1:** Time Exponent (*n*) and Oxidation Rate Constant (*k*_*p*_) Estimated Using eq 1 for Both the As-Printed and Aged Alloys at 750 and 900°C

alloy system	temperature	*n*	*K_p_* (g/cm^2^s^‑*n*^)
SLM as-printed	600°C	0.59	0.0002
	750°C	0.33	0.0016
	900°C	0.26	0.0060
SLM + aged	600°C	0.64	0.0003
	750°C	0.26	0.0039
	900°C	0.59	0.0043

In power-plant applications such as boiler tubes, components are generally exposed to lower temperatures (around ∼600°C),^57–59^ and are expected to sustain for a longer time. However, long-duration laboratory experiments are not feasible; therefore, oxidation degradation of materials is usually estimated using accelerated techniques, such as increasing the temperature, to attain measurable changes on the surface. Besides, increasing the temperature also enhances the energy efficiency of power plants. Inspired by this idea, material degradation at much higher temperatures was investigated in the current study. Images of the isothermal oxide layer thickness growth with time durations of 24, 48, 72, and 96 h at temperatures of 900°C, 1050°C, and 1200°C for both the as-printed and aged alloys are presented in Figure 5 (a,b), respectively. The images show that the oxide layers consist of a homogeneous compact outer oxide layer and an inhomogeneous inner oxide layer beneath it. It is also evident that the thickness of the duplex oxide layer (i.e., the combined thickness of the homogeneous and inhomogeneous layers) increases with time and temperature; however, the effect of temperature on the thickness growth variation appears to be more pronounced than that of time. It can also be observed that with increasing temperature, the growth of the inner inhomogeneous oxide layer is more dominant than that of the homogeneous oxide layer, indicating that oxygen diffusion-guided activity through the oxide layer is more dominant with increasing temperature. The initially formed homogeneous oxide layer acts as a protective barrier for element diffusion from the base alloy.

**Figure 5:**
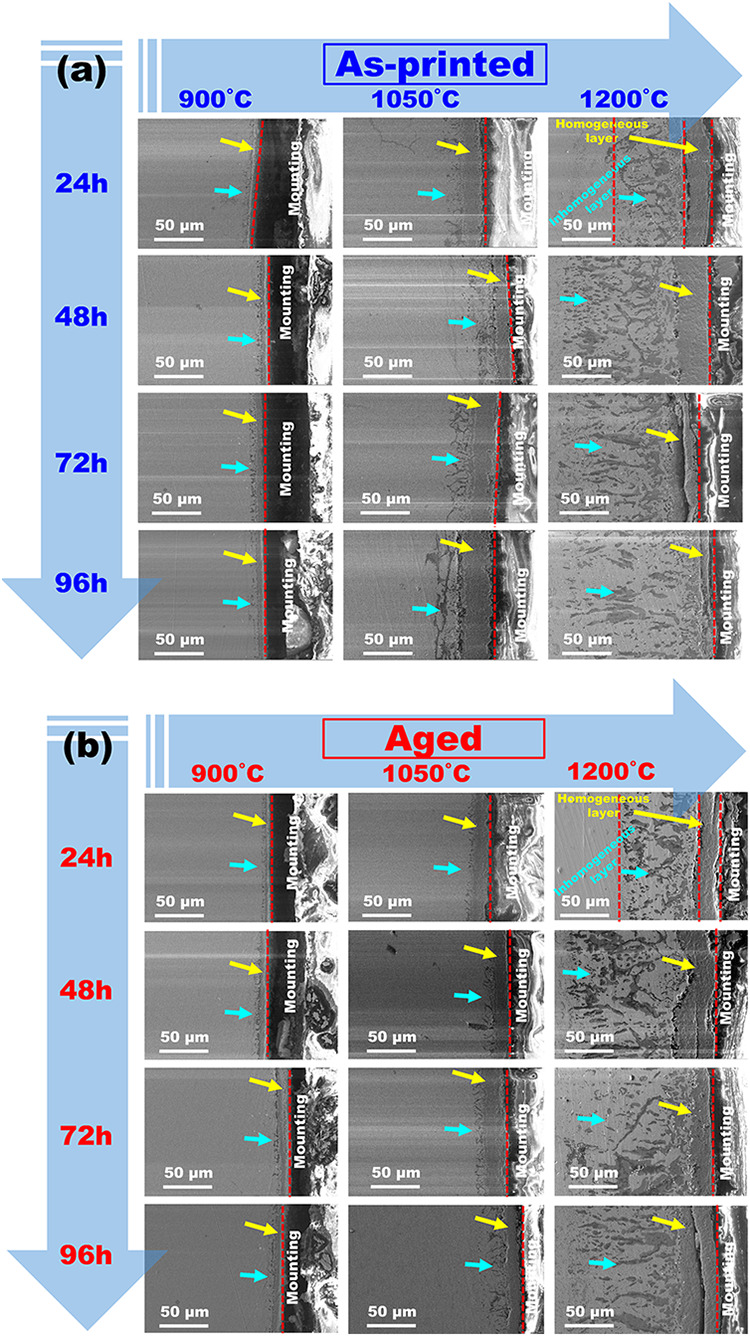
SEM cross-sectional view of the oxide layer formed on the surface subjected to oxidation for various times and temperatures in the (a) as-printed and (b) aged alloys.

The variation in the thickness of the duplex oxide layer with temperature and time for both alloys, estimated from the SEM images, is shown in Figure 6 (a). The oxide layer growth kinetics for both the as-printed and aged alloys follow a nearly parabolic growth trend. By applying a similar power law for oxide growth kinetics (eq 2), the rate constants (*k’*_*p*_) at different temperatures were evaluated and are listed in Table S1.

2 $$\Delta x=k_{p}’t^{n}$$

where Δ*x* is the thickness of the oxide layer. The parameters *t* and *n* are the same as those mentioned in eq 1. The estimated rate constants (in natural logarithm) vs the inverse of temperature (1/*T*) are plotted in Figure 6 (b). By applying linear regression to the estimated rate constants vs (1/*T*) plot, the activation energy was calculated using the following Arrhenius-type rate relation (eq 3).

3 $$k_{p}’=k_{0}e^{-Q/\mathrm{RT}}$$

where *k’*_*p*_ denotes the oxidation rate constant, *k*_0_ is the pre-exponential factor, *Q* is the activation energy, *R* is the universal gas constant, and *T* denotes the temperature in Kelvin. The estimated activation energy for oxidation (*Q*) and pre-exponential factor (*k*_0_) for the as-printed alloy are 154.07 kJ/mol and 22.74 × 10^6^ μm/h^0.5^, respectively. For the aged alloy, the *Q* and *k*_0_ values are 152.83 kJ/mol and 10.98 × 10^6^ μm/h^0.5^, respectively. The activation energies for both the as-printed and aged alloys are close to each other. This might be a consequence of the aging treatment of the as-printed alloy during the extended oxidation process. The similar oxidation activation energy values highlight the significance of the mechanical advantage of the nanoprecipitation-strengthened aged alloy over the as-printed alloy in terms of oxidation degradation. However, the pre-exponential factor is comparatively higher in the as-printed alloy, indicating the opposite effect with increasing temperature during oxidation.

**Figure 6:**
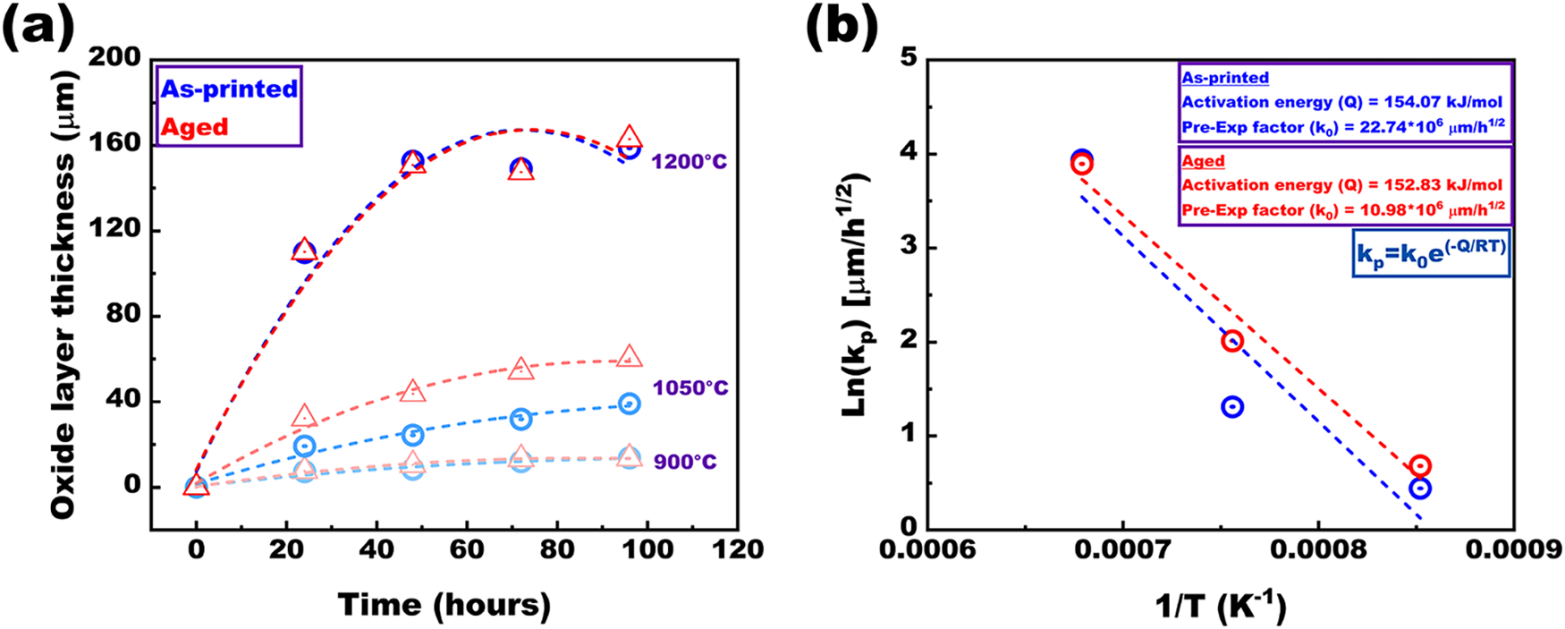
(a) Isothermal oxide layer growth variation with time and temperature for the as-printed (blue) and aged alloys (red). Dotted lines are parabolic fitted curve; (b) natural logarithm of oxidation growth rate constant *k*_*p*_ vs inverse of temperature plot, along with the linear regression (dotted line) curve considering the Arrhenius rate equation.

Although the oxide thickness exhibits near-parabolic growth at 900°C, the corresponding mass gain in the as-printed alloy follows subparabolic kinetics. This apparent deviation arises from the complex, multilayered nature of the oxide structure. The Pilling–Bedworth ratio of the predominantly formed Cr-oxide is approximately ∼2, indicating the formation of a dense and protective oxide scale (visualized in Figures 8 and 9). The oxide scale beneath the Cr-oxide layer is inhomogeneous and incomplete due to limited oxygen ingress through the Cr-oxide layer. This limited oxygen diffusion reduces the mass uptake while still contributing to the oxide thickness, which reasonably explains the observed deviation between the mass gain and thickness growth kinetics.

### 3.3 Oxide Product Investigation

Oxide products formed at 900°C, 1050°C, and 1200°C were investigated using XRD and Raman spectroscopy. The XRD patterns of both the as-printed and aged alloys, after 96 h of oxidation at all three temperatures, are presented in Figure 7 (a,b), respectively. The major oxide peaks, along with the primary fcc matrix peaks, are associated with Cr-oxide and Ti oxide. A few peaks related to other elemental oxides and mixed/spinel phases were also observed in the patterns. The Raman spectra of both the as-printed and aged alloys oxidized at different temperatures for 96 h are presented in Figure 7 (c–e) and (f–h), respectively. Similar to the XRD results, the major peaks in the spectra were associated with Cr and Ti oxides. Other minor elemental oxide peaks, as well as mixed/spinel oxide peaks, were also identified in the patterns. The results indicate that all elements underwent oxidation, with Cr and Ti oxidizing predominantly due to their higher oxidizing propensity.^60^ Overall, the observations indicate that the major fraction of oxides formed is related to Cr and Ti oxides.

**Figure 7:**
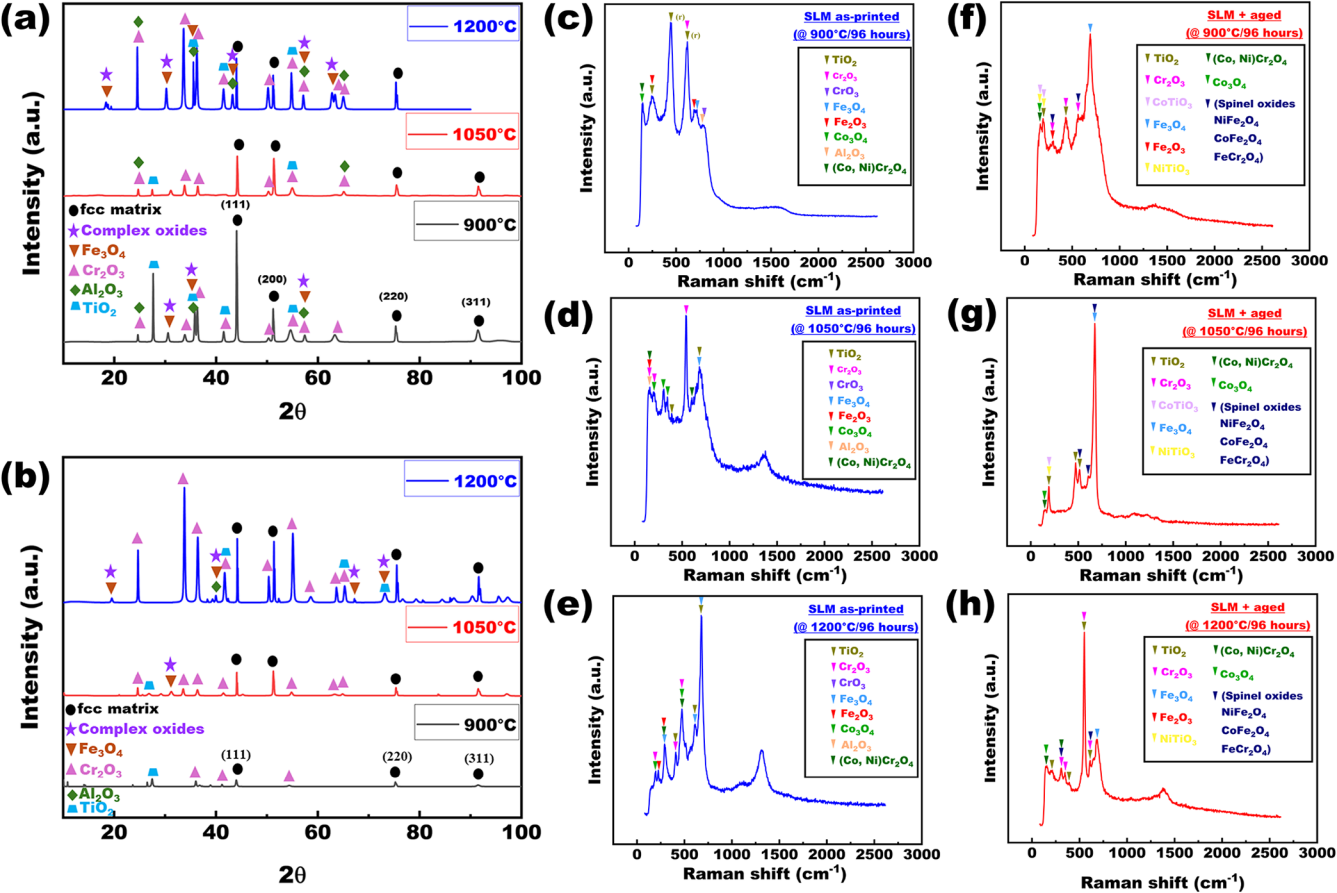
(a, b) XRD patterns of the as-printed and aged alloys after 96 h of isothermal oxidation, respectively. (c–e) Raman spectrum of the as-printed alloy oxidized at 900°C, 1050°C, and 1200°C, respectively. Similarly, (f–h) show the Raman spectrum of the aged alloy oxidized at 900°C, 1050°C, and 1200°C, respectively.

The oxide layers formed at 900°C, 1050°C, and 1200°C were also characterized using SEM-EDS elemental mapping. The EDS elemental mappings of the oxide layer formed at 1050°C for both the as-printed and aged alloys are presented in Figures 8 and 9, respectively. The elemental mapping formed at 1050°C was taken as a reference for explanation, and the similarities, as well as the differences, observed at other temperatures are highlighted accordingly. The EDS elemental maps show that the top section of the homogeneous oxide layer predominantly consists of the Ti, Fe, Ni, and Co elements with a very minor amount of Al and Cr. This indicates the formation and coexistence of elemental oxides as well as mixed/spinel oxides and corroborates well the XRD and Raman spectroscopy results. The second section (layer below the top section) of the homogeneous layer showed the presence of predominantly Cr, indicating the formation of a protective Cr-oxide layer. The inner inhomogeneous layer is an Al and Ti oxide layer. The corresponding point EDS results presented in Figures S3 and S4 also confirm the observation of multiple oxide layers. A similar elemental distribution in the oxide layers in both the alloys was observed at 900°C and 1200°C (presented in Figures S5 to S8), with the only difference being the thickness of the oxide layer (both homogeneous and inhomogeneous layers). The oxide layer thickness is comparatively low at 900°C, but significantly higher at 1200°C. With increasing time and temperature, the thickness of the Cr-oxide layer increases, which indicates the dominant outward diffusion of Cr from the bulk as compared to the other elements after the development of the protective Cr-oxide layer. The thickness of the inhomogeneous layer beneath the Cr-oxide layer also increases with time and temperature, suggesting the diffusion of oxygen through the Cr-oxide layer toward the bulk and the preferential formation of Al and Ti oxides due to their higher oxidizing propensity compared to that of the other elements.

Usually, Al_2_O_3_ and Cr_2_O_3_ layer formation has been reported to act as protective layers against oxygen diffusion into the base alloy. The oxide layer characterization results demonstrate the formation of a stable and protective Cr_2_O_3_ layer over time. The formation of the Cr_2_O_3_ layer leads to the gradual suppression of the oxidation rate and results in a subparabolic kinetics profile. The weight gain in the aged alloy is comparatively higher than in the as-printed alloy due to the presence of nanoprecipitates, which influences the formation of the protective layer. In the as-printed condition, Cr is evenly distributed, which assists in the earlier and easier formation of the Cr-oxide protective layer. Meanwhile, the precipitates are deficient in Cr in the aged alloy; hence, the formation of an effective Cr-oxide protective layer is comparatively lagging.^55,56^ Apart from the uneven distribution of Cr, the additional matrix–precipitate phase boundaries in the aged alloy can provide short-circuit channels for oxygen diffusion, which exacerbates the oxidation process.^61^ It is well known that the oxidation process in metals and alloys is mediated and controlled by the inward and outward diffusion of oxygen and metal ions through the initially formed oxide scales. Similar oxide layer formation and diffusion-controlled oxidation mechanisms have also been highlighted in HEAs.^33,62–64^ In the selected HEA system, at lower temperatures, typically up to 900°C, the formation of a Cr-oxide layer sufficiently protects the alloy base from further oxidation by preventing oxygen diffusion in both alloys. Therefore, the inhomogeneous oxide layer beneath the Cr-oxide layer is not very significant, as presented in Figures 8 and 9 as well as in Figures S5–S8. However, the oxide scale reflects oxygen diffusion through the Cr-oxide layer, probably by microcracks on the layer and grain boundary channels.^65^ At higher temperatures, the oxygen diffusion rate is more rigorous, and a significant inhomogeneous oxide layer is formed. This observation indicates the defects and instability of the Cr-oxide layer as well as the obvious temperature-dependent activity of the occupied species with increasing temperature. The comparative outward diffusion of only Cr and the thickening of Cr-oxide despite Al and Ti are due to differences in their atomic sizes, which have been described in the later section explaining the mechanistic aspect of oxide layer growth.

**Figure 8:**
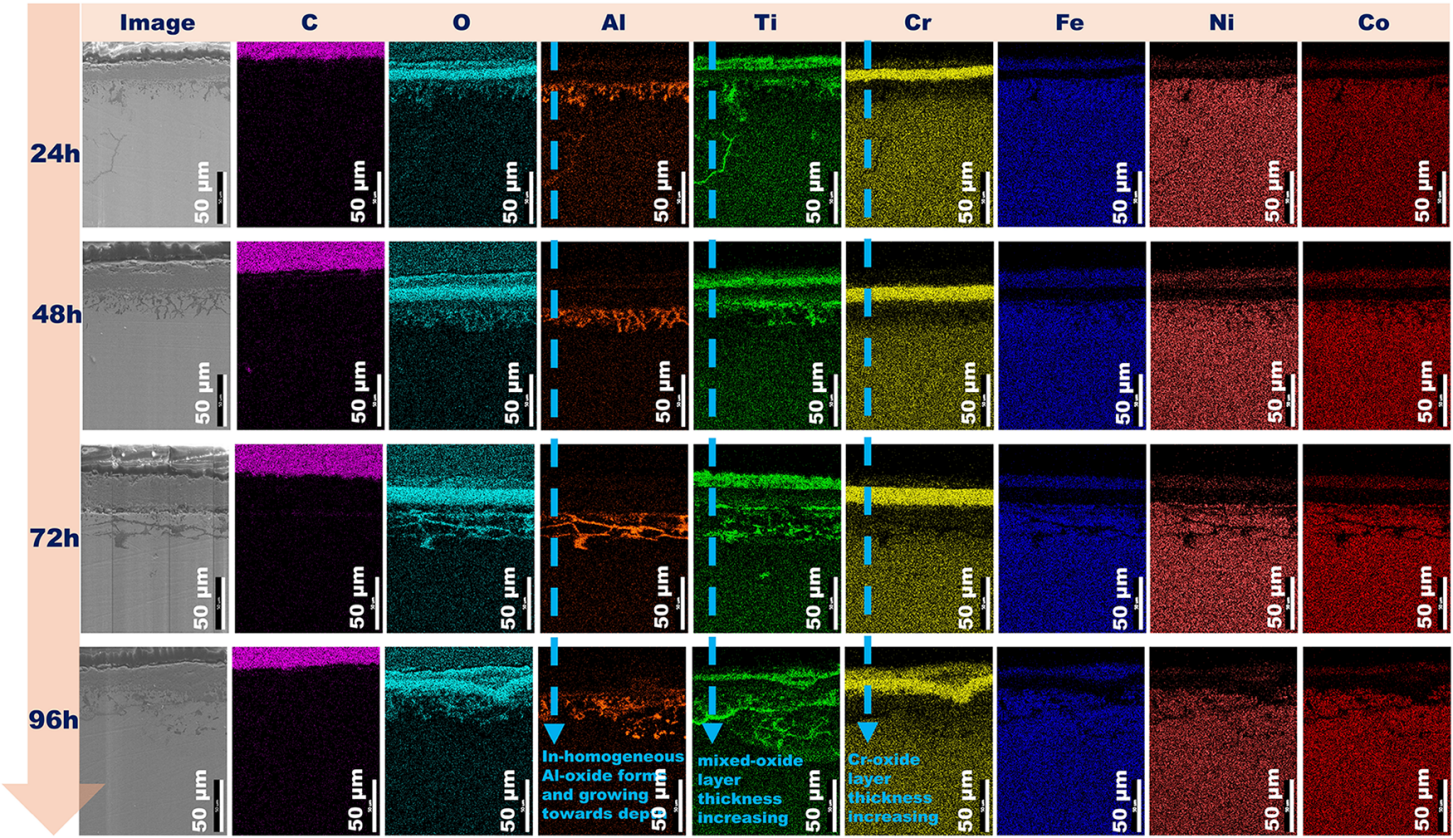
Cross-sectional EDS elemental mapping of the as-printed alloy oxidized at 1050̊°C.

**Figure 9:**
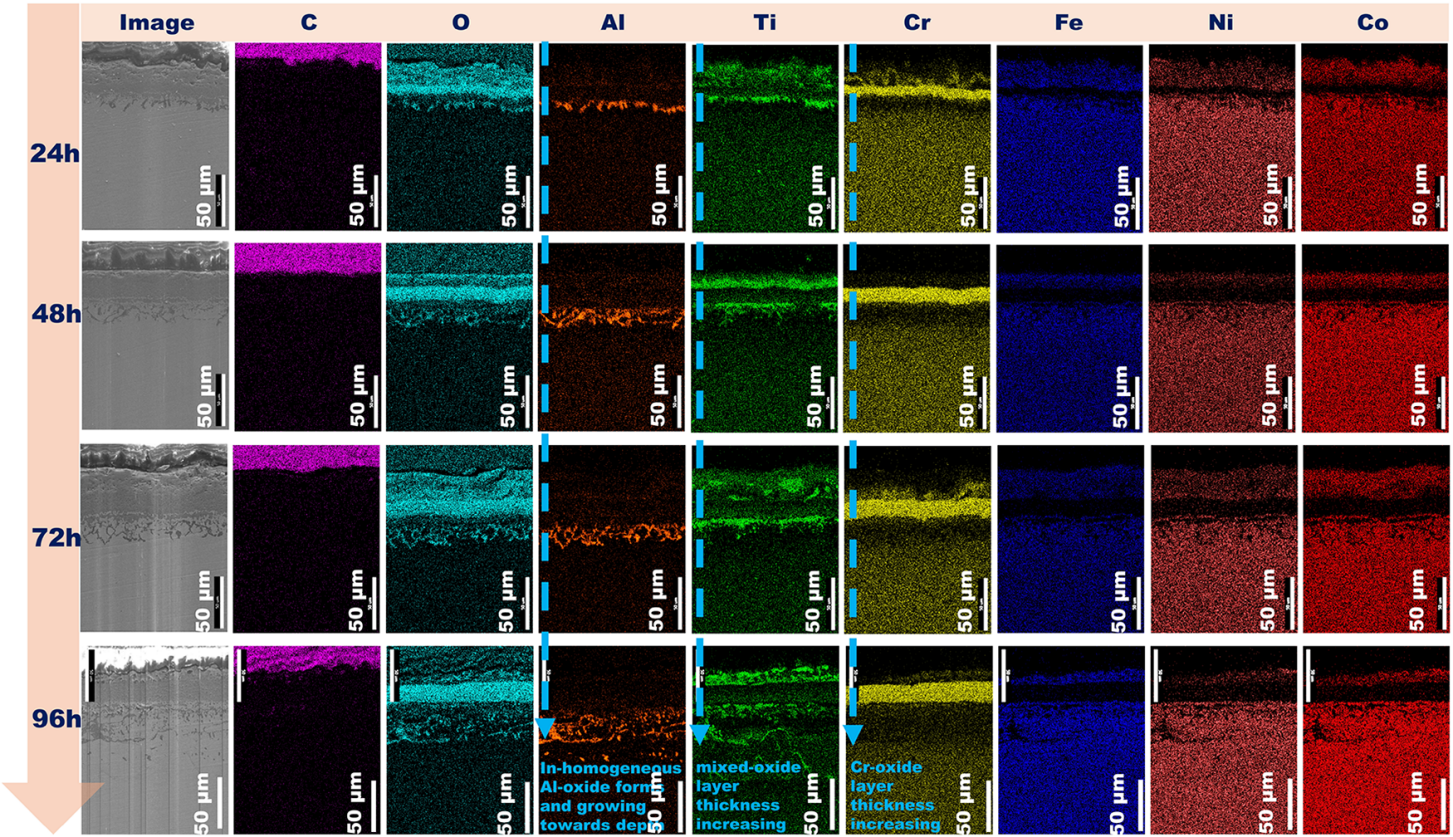
Cross-sectional EDS elemental mapping of aged alloy oxidized at 1050°C.

### 3.4 Mechanistic Aspects of Oxide Layer Formation

A mechanistic understanding of oxide formation and oxide scale distribution is guided by the interactive tendency of species with the external environment (thermodynamic criteria) and the processes involving the mobility of species toward the oxide/metal interface, transfer across the oxide/metal interface, and channels across the oxide layer. Overall, surface speciation is influenced by factors such as elements’ reactivity with oxygen (i.e., standard reduction potential, (E^0^) and perturbation of element mobility (i.e., atomic size (I) and cohesive energy density (CED)). The combined aspect of these parameters delineates the feasibility and propensity of oxide layer formation, referred to as the preferential interactivity parameter (PIP) map.^66,67^ The PIP map for the elements contained in the selected alloy is presented in Figure 10 (a), and the corresponding values are tabulated in Table S2, taken from an open source. The results obtained using EDS, XRD, and Raman spectroscopy suggest that each element in the system is initially exposed to the environment and undergoes oxidation during the initial period. The oxidation of elements leads to the formation of individual metallic oxides as well as mixed and complex/spinel oxides at the top surface, as detected in the Raman spectrum and XRD. The outermost layer, constituting the oxide of every element, as observed in the cross-sectional EDS maps as well as top surface EDS maps presented in Figure S9 (as-printed) and Figure S10 (aged), also confirms the oxide formation. The covered oxide layer hinders the oxidation process, and the preferential interactive parameters (i.e., redox-driven oxidation propensity (E^0^) and diffusion controlling parameters r and CED), govern the enhanced oxidation activity of the elements with time. Among the constituting elements, Al, Ti, and Cr exhibit comparatively lower E^0^ values. Although Cr possesses a comparatively higher reduction potential than Al and Ti, its smaller atomic size favors easier mobility, leading to dominant outward diffusion and the formation of a continuous Cr-oxide layer. Moreover, the diffusion behavior can also be rationalized based on the activation energy and ionic valence, as described in eqs 4 and ([Disp-formula eq5]), respectively.

4 $$D=D_{0}e^{\left(-\Delta E_{a}/\mathrm{kT}\right)},\left\{D\propto e^{\left(-\Delta E_{a}/\mathrm{kT}\right)}\right\}$$

5 $$D=\frac{\mu \mathrm{kT}}{\mathrm{Ze}},\left\{D\propto \frac{1}{Z}\right\}$$

where *D*, Δ*E*_*a*_ *Z*, and *e* are the diffusion coefficient, activation energy barrier, valence of thee diffusing species, and electron charge, respectively.

**Figure 10:**
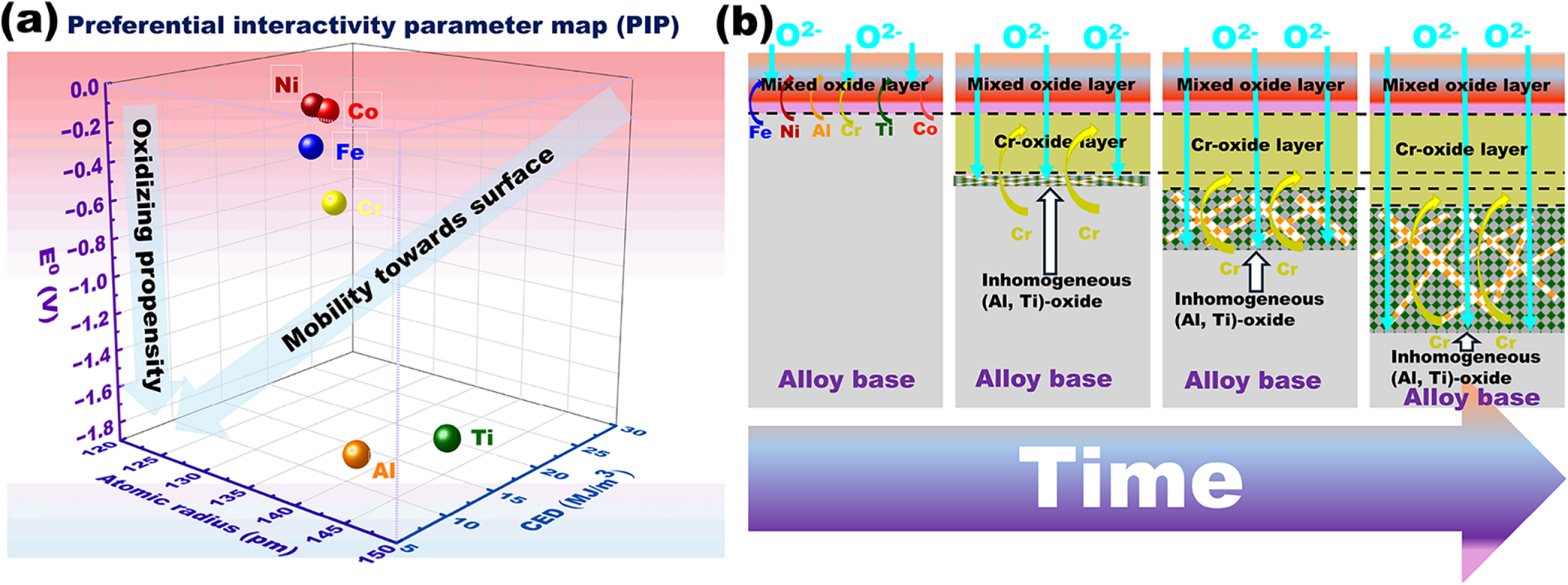
(a) Preferential interactivity parameter map for the elements contained in the selected alloy system, highlighting the oxidation activity of elements. (b) Schematic of oxide layer growth over time.

Elements such as Al and Ti exhibit higher activation energies and higher valence states (Al^3+^ and Ti^4+^), resulting in lower diffusion coefficients. In contrast, Cr exhibits a comparatively favorable diffusion behavior than Al and Ti, enabling the formation of a protective Cr-oxide layer. The relatively low concentrations of Al and Ti in the alloy system (inset table in Figure 2) also prevent the formation of a continuous oxide layer. The formed Cr-oxide layer envelopes the alloy base and restricts element diffusion. Simultaneously, due to the higher oxidizing propensity (according to the PIP map), inhomogeneous Al and Ti oxides formed under the Cr-oxide layer. Over time, due to several possibilities such as microcracks in the oxide layer and other channels, oxygen diffused across the Cr-oxide layer and preferentially oxidized Cr, Al, and Ti. As a consequence of similar oxidation phenomena, the Cr-oxide layer thickens, and inhomogeneous Al and Ti oxides formed beneath the Cr-oxide layer intrude inside the base with extending time. The preferential outward diffusion of Cr compared to other elements has also been observed in similar complex alloy systems, leading to the formation of a continuous Cr_2_O_3_ layer, while other elements form mixed oxides.^46,53,54^ Thermodynamically and kinetically, other elements such as Ni, Co, and Fe are positioned in the PIP map (Figure 10(a)) at less likely positions to oxidize. The oxidation process over time is schematically shown in Figure 10(b).

## 5 Conclusions

Motivated by the mechanical significance of additively manufactured Al_0.2_Co_1.5_CrFeNi_1.5_Ti_0.3_, the present study explored its isothermal oxidation behavior in both the as-printed state with a single phase and in the aged state consisting of nanoprecipitates. The primary aim of this study is to assess the high-temperature sustainability of these alloys for applications in gas turbines, power plants, and the aerospace sector. The kinetics perspectives, oxide layer characterizations, and mechanistic aspects of oxide layer formation have been extensively explored. The analysis and findings of this study reached several conclusions, which are summarized below:

(1) The weight gain kinetics below 900°C follow subparabolic oxidation kinetics, shifting toward parabolic kinetics with increasing temperature. Oxide growth above 900°C attained nearly parabolic kinetics.
(2) The oxide layer consists of three different layers. The outermost elemental as well as mixed/spinel oxide layers, followed by a homogeneous Cr-oxide layer, and finally inhomogeneous Al and Ti oxide layers. A homogeneous Cr-oxide layer acted as a protective layer and significantly suppressed the oxidation activity by restricting the diffusion of oxygen and metal ions.
(3) Homogeneous Cr-oxide layer and inhomogeneous Al/Ti oxide layer growth are more sensitive to temperature than to time. This highlights the better protective nature of the Cr-oxide layer.
(4) The combined impact of the standard reduction potential, size factor, and CED guided the oxide layer formation. The impact of the dominant size factor leads to the formation of an outer Cr-oxide layer instead of Al and Ti oxide layers, despite the higher oxidizing potential.
(5) The comparative aspect of the mechanical and oxidation behaviors demonstrates that the nanoprecipitation-strengthened aged alloy offers a mechanical advantage over the as-printed alloy, without significant oxidation degradation at elevated temperatures.

## Supplementary Material



## Data Availability

All data are available in the main text or the Supporting Information.

## Notes

Additional experimental results, such as visual images, SEM-EDS, and tables containing estimated values and schematic details of oxidation experiments (PDF)
