# Supplementary material for: Delineating Oxidation Aspects of an Additively Manufactured Nanoprecipitation-Strengthened Al0.2Co1.5CrFeNi1.5Ti0.3 High-Entropy Alloy
Source: ACS Appl Nano Mater. 2026 Jun 2;9(23):10544–55. doi: 10.1021/acsanm.6c00394 (PMC13270461; doi:10.1021/acsanm.6c00394)
Supplement: Supplementary file 1 [file an6c00394_si_001.pdf]

Supporting Information

Delineating oxidation aspects of additively manufactured nanoprecipitation strengthened $Al_{0.2}Co_{1.5}CrFeNi_{1.5}Ti_{0.3}$ high entropy alloy

Poresh Kumar,[1,2,3,4], Tu-Ngoc Lam,[2,4,5] Po-Heng Chou,[4,7] An-Chou Yeh,[4,7] Peter K. Liaw,[6] E-Wen Huang[2,4*] and Sudhanshu Shekhar Singh[3*]

[1] *International College of Semiconductor Technology, National Yang Ming Chiao Tung University, Hsinchu, 30010, Taiwan*

[2] *Department of Materials Science and Engineering, National Yang Ming Chiao Tung University, Hsinchu, 30010, Taiwan*

[3] *Department of Materials Science and Engineering, Indian Institute of Technology, Kanpur 208016, India*

[4] *High Entropy Materials Center, National Tsing Hua University, Hsinchu, 30013, Taiwan*

[5] *Cuu long University, Vinh long province 890000, Vietnam*

[6] *Department of Materials Science & Engineering, The University of Tennessee, Knoxville, 37996-2100 USA*

[7] *Department of Materials Science and Engineering, National Tsing Hua University, Hsinchu, 30013, Taiwan*

* Correspondence: ewenhuang@nycu.edu.tw; sudhanss@iitk.ac.in

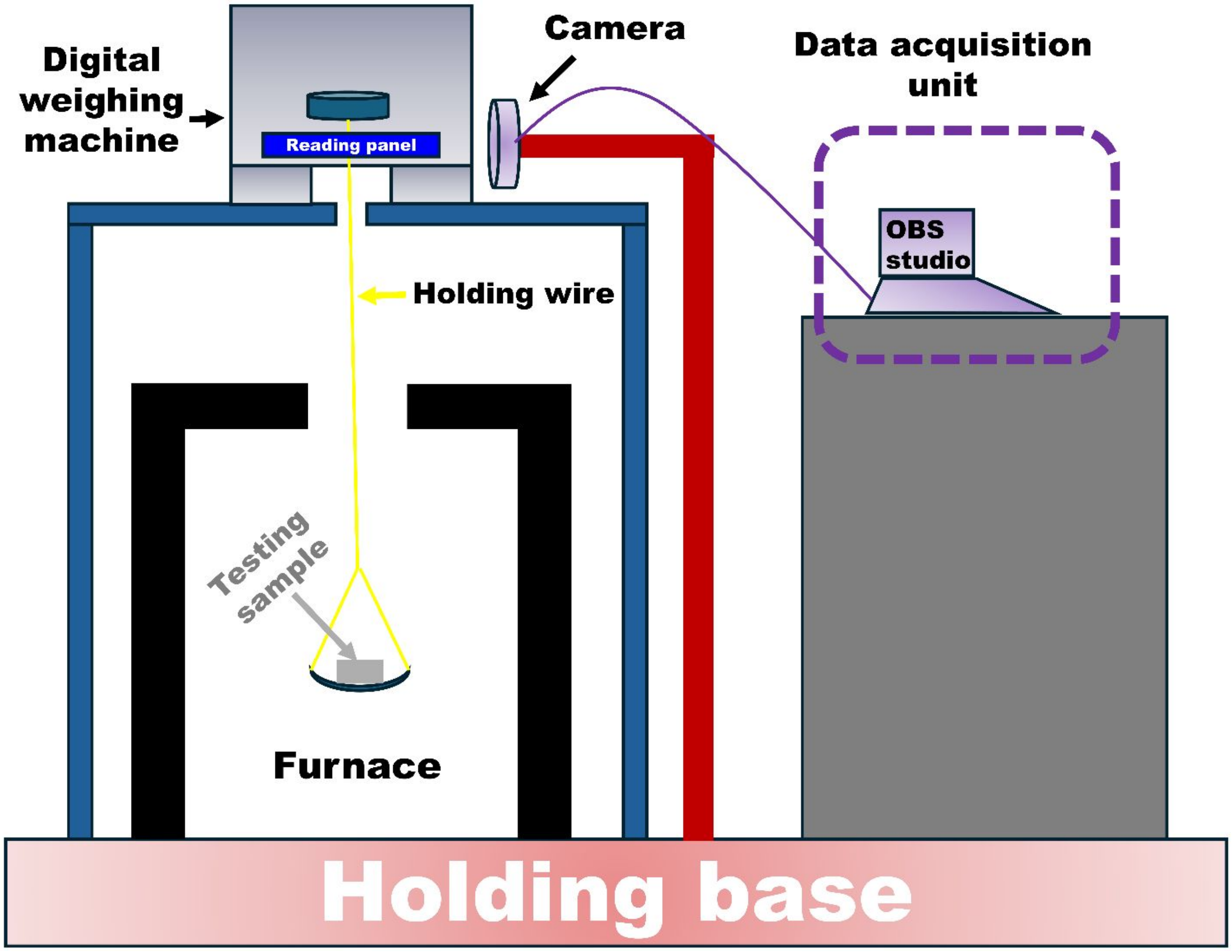

Figure S1: Schematic of oxidation experiment set up equipped with integrated weighing machine.

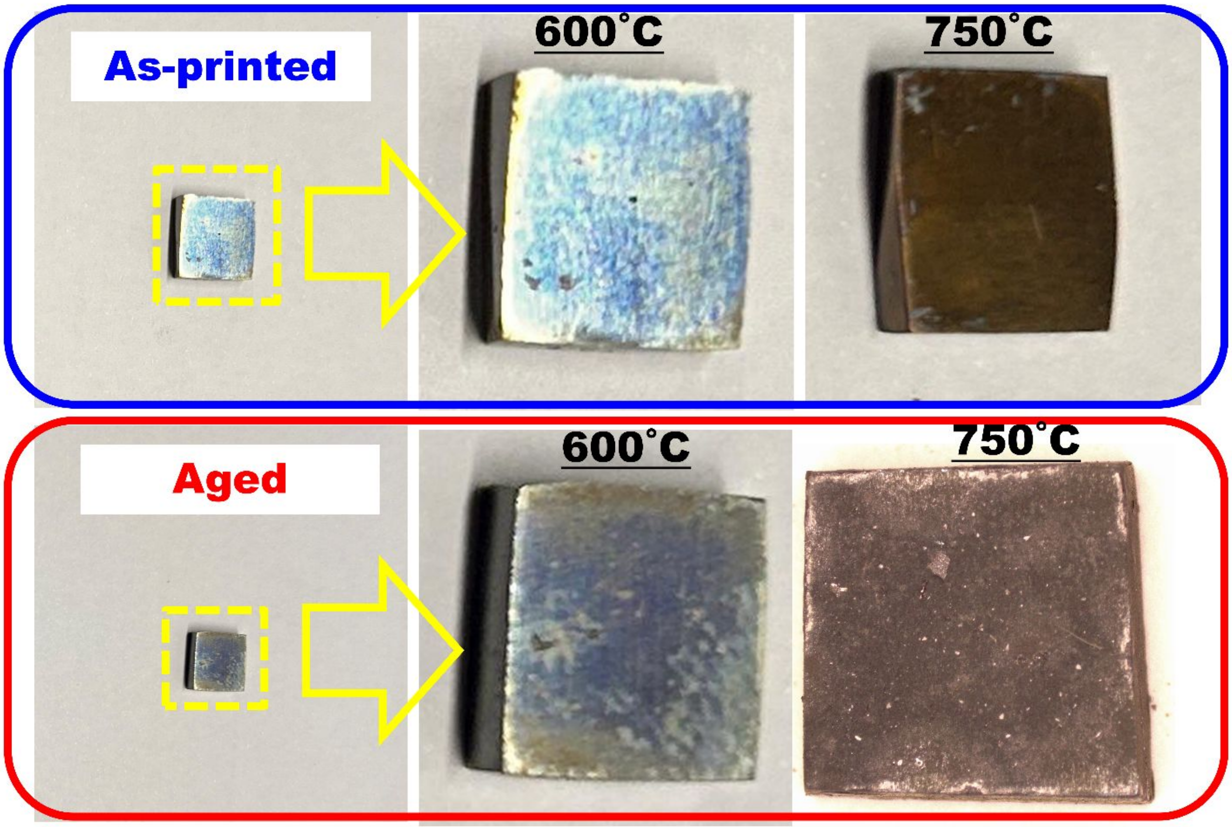

Figure S2: Visual camera image of surface oxide layer at 600˚C and 750˚C.

Table S1: The estimated oxidation rate constant (k_p) for both the as-printed and aged alloys at different temperatures.

Alloy system	Temperature	k_p (μm/h$^{0.5}$)
SLM as-printed	900 ˚C	0.192
	1050 ˚C	0.568
	1200 ˚C	1.709
SLM + aged	900 ˚C	0.296
	1050 ˚C	0.875
	1200 ˚C	1.692

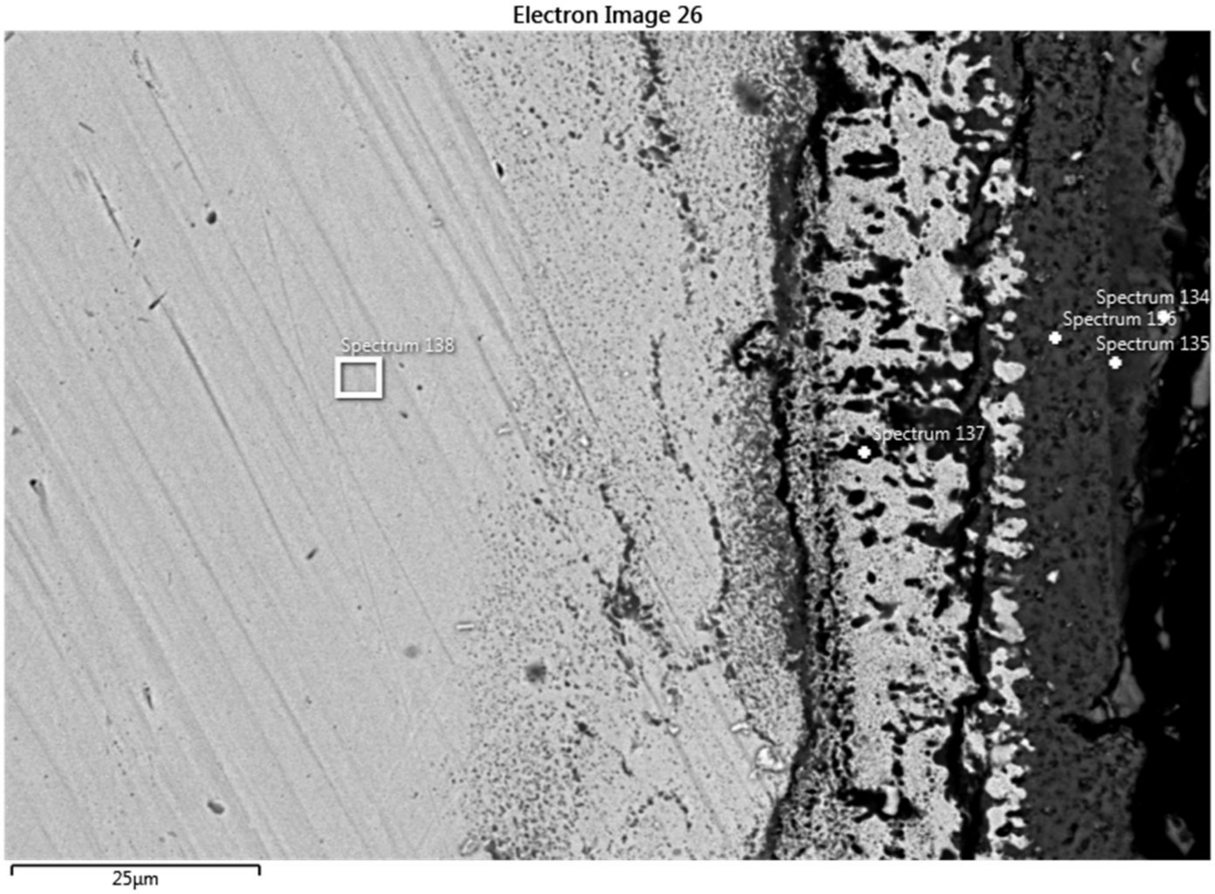

Spectrum Label	Spectrum 134	Spectrum 135	Spectrum 136	Spectrum 137	Spectrum 138
O	25.78	34.17	67.51	12.27	4.34
Al	12.03	2.61	0.09	1.24	3.11
Ti	1.07	2.09	2.76	12.15	4.08
Cr	3.04	15.38	28.38	10.98	17.45
Fe	16.57	13.24	0.64	16.45	17.10
Co	21.70	17.16	0.28	24.52	27.65
Ni	19.81	15.36	0.35	22.39	26.28
Total	100.00	100.00	100.00	100.00	100.00

Figure S3: Point EDS at different locations of the oxide scale for as-printed alloy oxidized at 1050 ˚C.

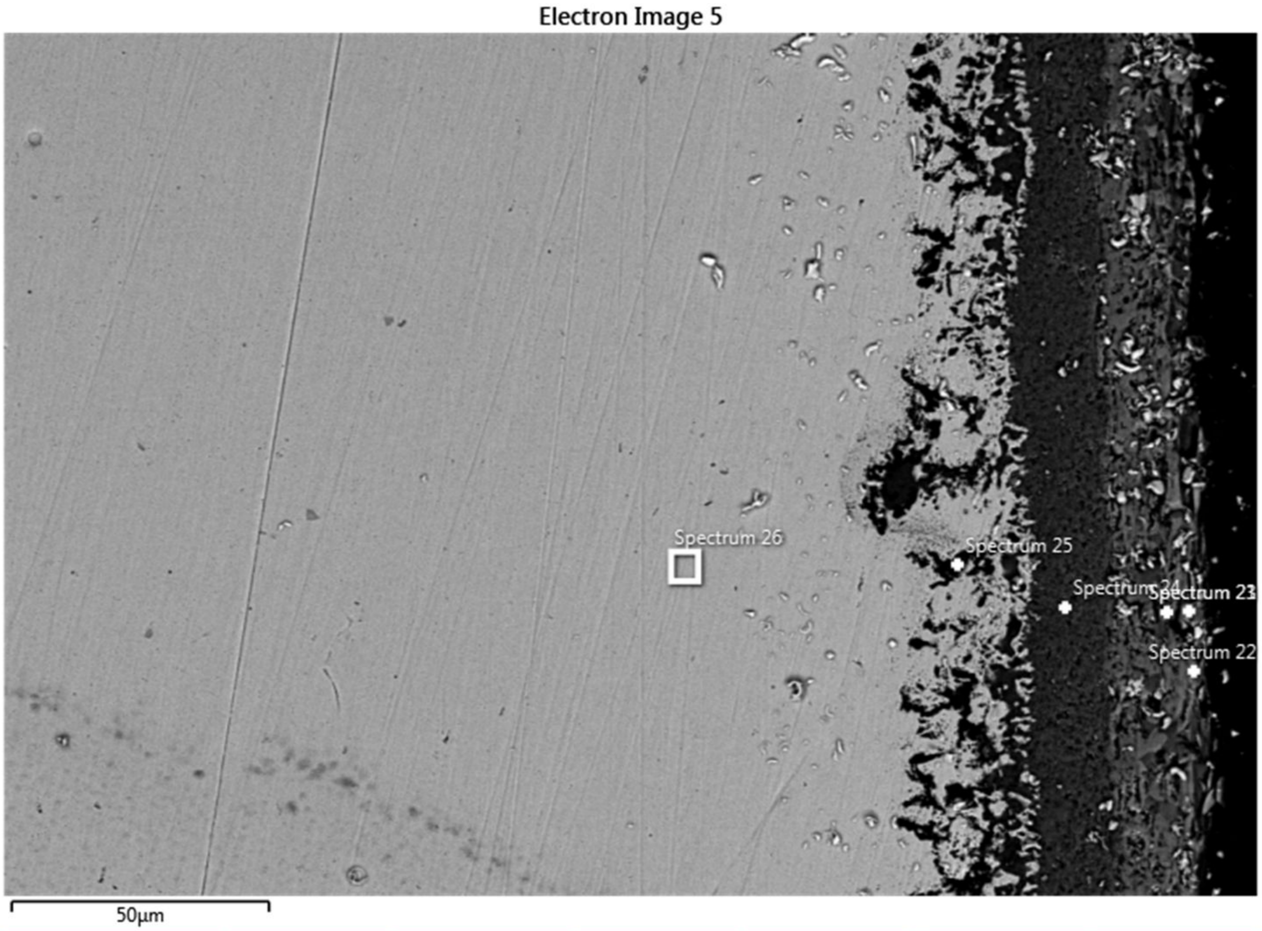

Spectrum Label	Spectrum 21	Spectrum 22	Spectrum 23	Spectrum 24	Spectrum 25	Spectrum 26
O	57.38	64.66	41.56	49.35	5.66	4.05
Al	0.70	1.04	1.06	2.24	0.54	3.27
Ti	8.53	6.73	8.33	5.78	2.66	4.09
Cr	1.32	1.51	7.39	21.22	10.49	17.45
Fe	10.54	8.62	15.22	2.08	21.03	17.07
Co	13.51	10.80	14.09	1.09	31.12	27.80
Ni	8.02	6.64	12.35	4.24	28.49	26.28
Total	100.00	100.00	100.00	100.00	100.00	100.00

Figure S4: Point EDS at different locations of the oxide scale for aged alloy oxidized at 1050 ˚C.

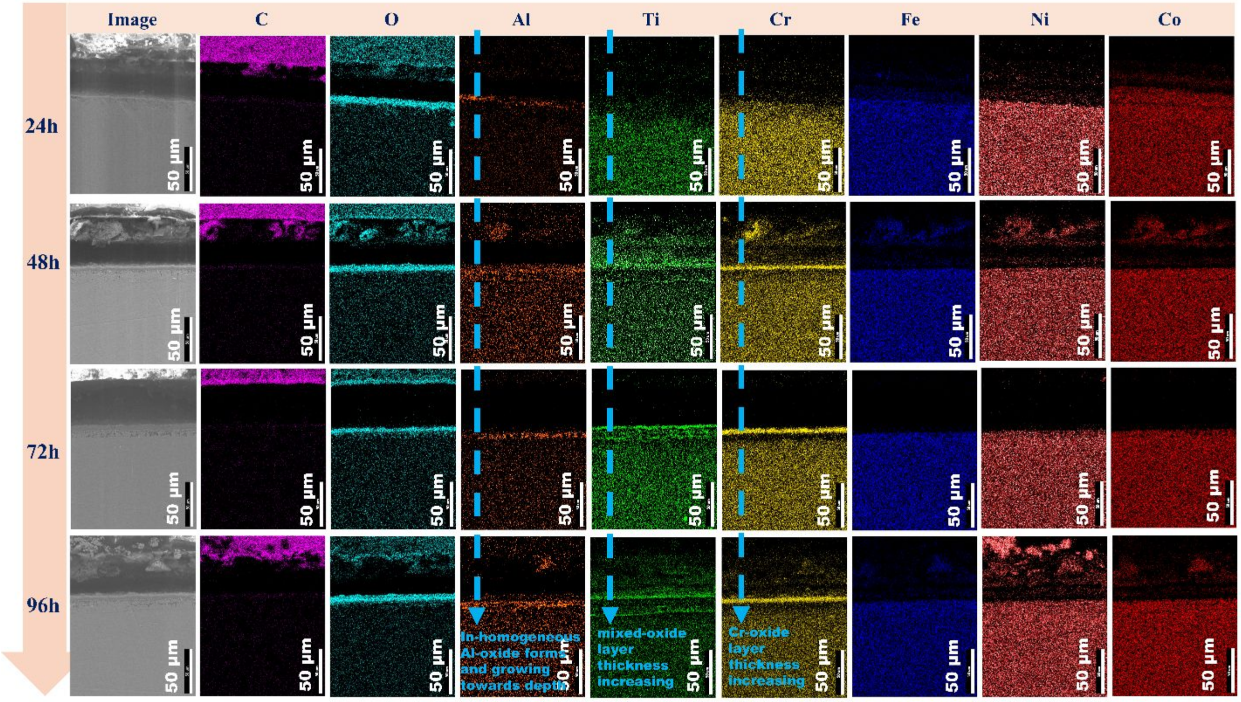

Figure S5: Cross sectional EDS elemental mapping of as-printed alloy oxidized at 900˚C

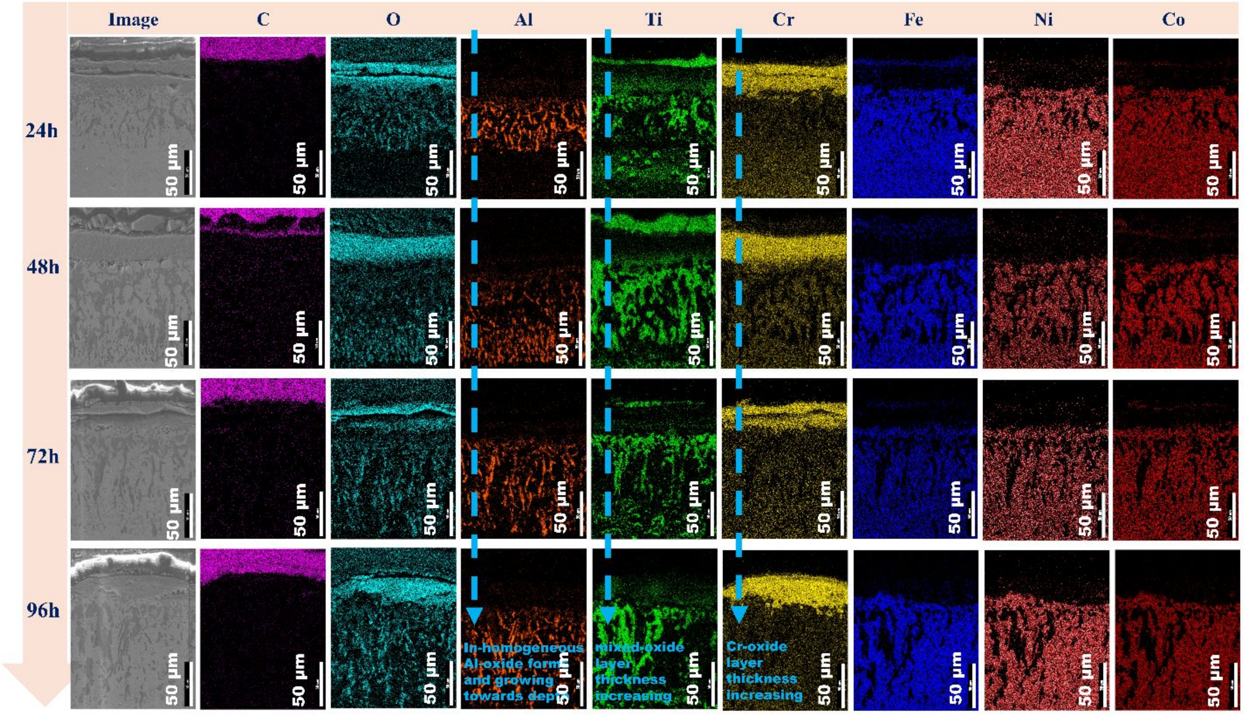

Figure S6: Cross sectional EDS elemental mapping of as-printed alloy oxidized at 1200˚C

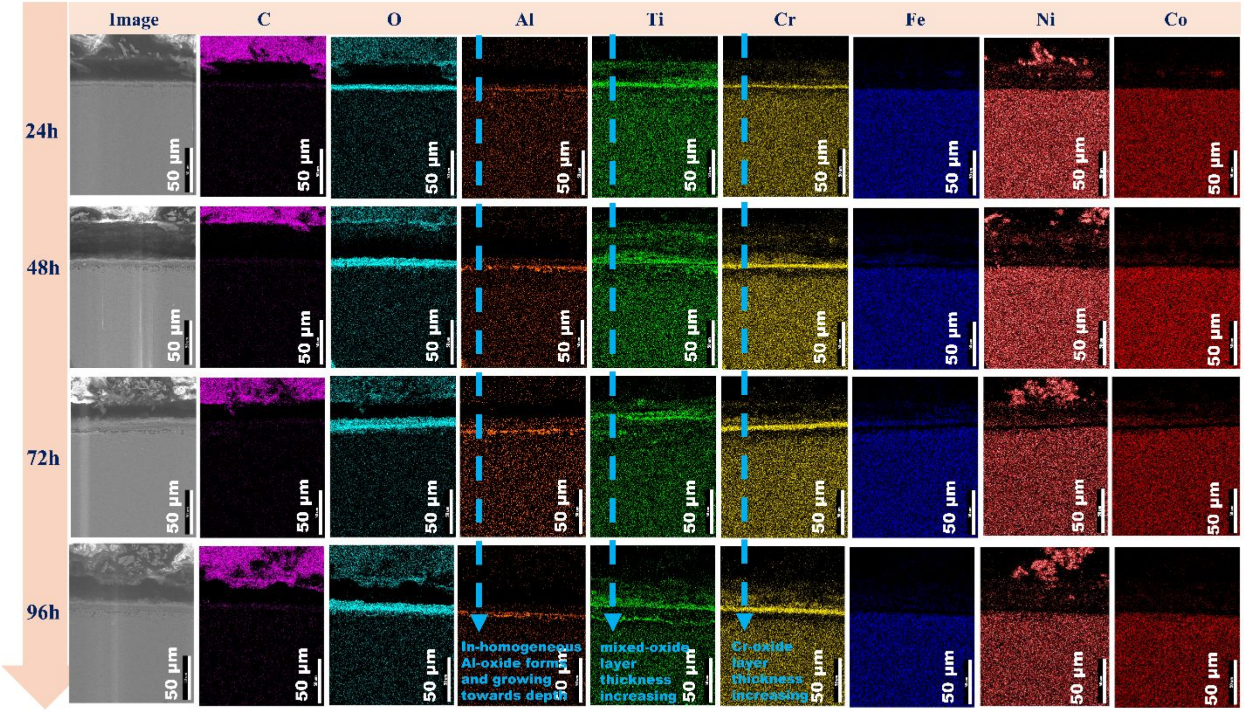

Figure S7: Cross sectional EDS elemental mapping of aged alloy oxidized at 900˚C

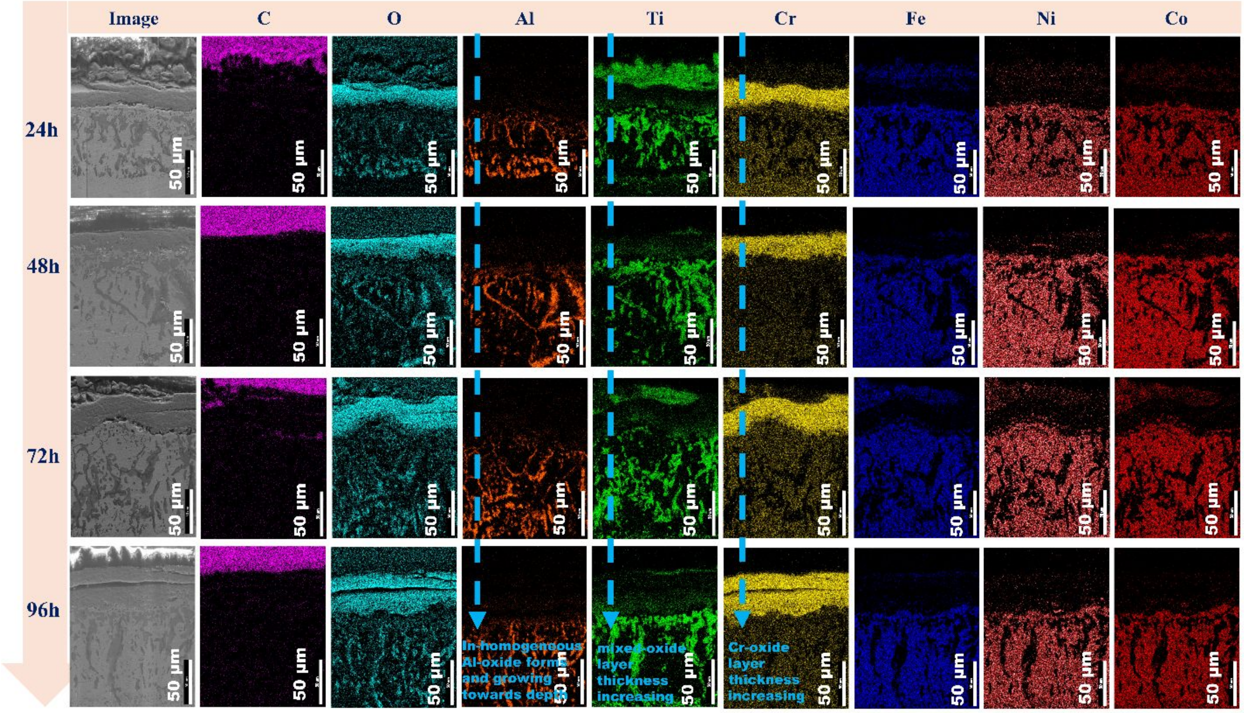

Figure S8: Cross sectional EDS elemental mapping of aged alloy oxidized at 1200˚C

Table S2: Values of parameter used to plot preferential interactivity parameter map (taken from open source google search).

Elements	Al	Co	Cr	Fe	Ni	Ti
**Standard reduction potential, E^0 (V)**	-1.66	-0.28	-0.74	-0.44	-0.25	-1.63
**Atomic size, I (pm)**	143	125	128	126	124	47
**Cohesive energy density, CED (MJ/m^3)**	8.1	22.3	20.2	19.6	21.7	13.2

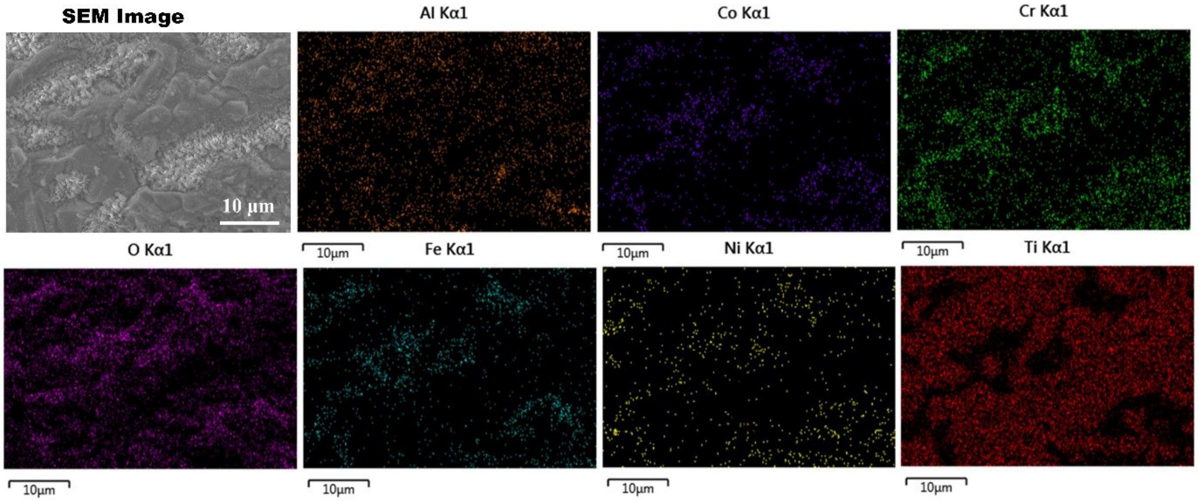

Figure S9: Top surface EDS elemental mapping of as-printed alloy oxidized at 1200˚C.

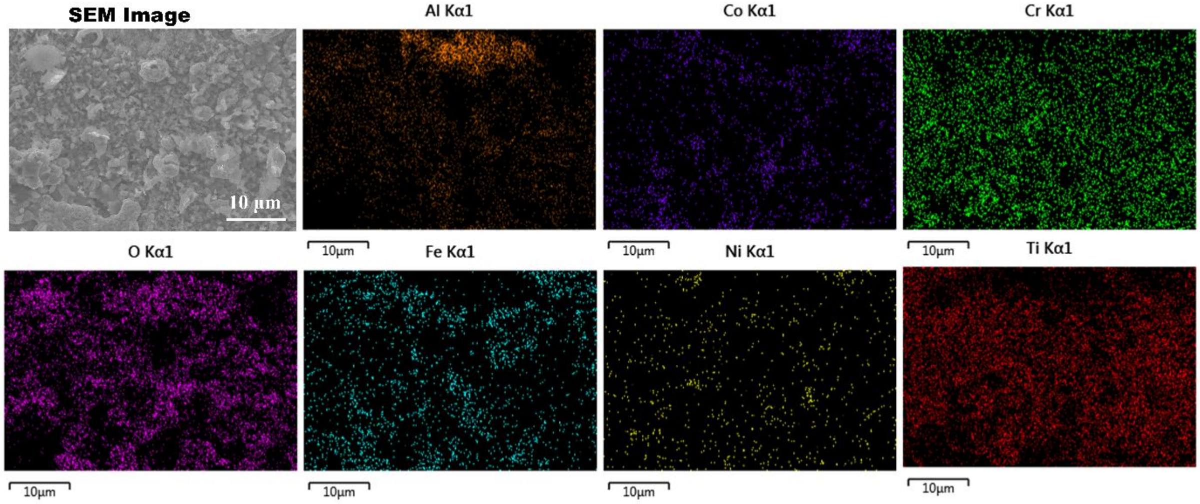

Figure S10: Top surface EDS elemental mapping of aged alloy oxidized at 1200˚C.
